# A New Screening Tool for Rapid Diagnosis of Functional and Environmental Factors Influencing Adults with Intellectual Disabilities

**DOI:** 10.3390/diagnostics12122991

**Published:** 2022-11-29

**Authors:** Nophar Ben David, Meir Lotan, Daniel Sender Moran

**Affiliations:** 1 Amichai Nonprofit Organization, Hod Hasharon 4510801, Israel; 2 Department of Physiotherapy, Faculty of Health Science, Ariel University, Ariel 40700, Israel; 3 Department of Health Systems Management, Faculty of Health Sciences, Ariel University, Ariel 40700, Israel

**Keywords:** intellectual disabilities, adults, functional and environmental changes, diagnosis of level of ID, COVID-19

## Abstract

The dynamic nature of intellectual disability (ID) and its many comorbidities necessitate repeated screening and functional diagnosis. However, the existing scales are lengthy and expensive and cannot be implemented at all levels of ID. During phase one of this study (September 2019–September 2020), the functional screening tool (FST-ID) was validated for adults with ID and then used as a clinical tool to collect data for 76 participants from their direct service providers (DSP). Their age ranged from 21 to 71 years (mean = 37.63, SD ± 14.61), and they were diagnosed with severe/profound (N = 16) and moderate (N = 29), and mild (N = 31) levels of ID. The scale was found to hold high psychometric values. During phase two (September 2020–June 2022), the scale was found to be reliable as a continuous, clinical on-going screening tool, enabling the screening of functional and environmental changes experienced by adults with ID during acute times of the pandemic and regular times alike. All 76 adults experienced changes at the senior’s daycare center mostly. The new scale may also help in the future to determine whether those changes only relate to post COVID-19 symptoms or other comorbidities as well.

## 1. Introduction

Intellectual and developmental disability (ID) is an umbrella term for a group of disorders that are usually presented at birth and negatively affect the trajectory of the individual’s physical, intellectual, and/or emotional development (approximately 2% of the world’s total population). The diagnosis includes IQ levels lower than 70 [1]. This population is usually divided into four functional levels: 69–55 (mild), 54–40 (moderate), 39–25 (severe), and <25 (profound) [2,3,4].

According to the biopsychosocial model developed by the World Health Organization (WHO) and other significant organizations relying on the “International Classification of Functioning, Disability and Health” (ICF), ID is assumed to be a dynamic phenomenon that changes over time. This assumption derives from the understanding that the functional abilities of an individual with ID can improve or deteriorate throughout their lifespan due to the individual’s health factors and external influencers. These influencing factors include personally customized programs, accessibility to appropriate healthcare services, and environmental facilitators/boundaries enabling the activity and participation of the individual with ID [1,5,6,7,8]. Therefore, to improve care for this group of service recipients, a reliable diagnostic tool providing reliable and updated information about individual functional abilities and environmental influences should be utilized.

Functional abilities include three main domains according to the accepted definitions: conceptual (communication and academic skills), practical (independent and everyday life skills), and social (social and interpersonal abilities) [2,9,10]. Individuals with ID, especially those diagnosed at the severe/profound (S/P) levels, who lack appropriate assessment tools tailored to their unique and numerous communicational, physical, and social accessibility needs [4,11], are generally at a high risk for functional and health-related problems. Although this population presents excess healthcare needs compared to the general population, they have limited access to regular healthcare services [2,10,12,13]. These predicaments lead to inequality in healthcare, impact the quality of life of the individual with ID, and burden direct service providers (DSP) [14]. When considering the assessment of the functional abilities of individuals, DSPs can recognize even small changes in their service recipients due to their daily and consistent interaction with these individuals [2,10,12,15]. Therefore, the need to consult with DSP when assessing and introducing therapeutic changes [10,16] by using appropriate data collecting tools is recognized.

While reviewing available tools for assessing or screening the functional state of adults with ID, we found that most of them were developed specifically for children or adults with mild IDs. Several that targeted other populations, such as neurotypical adults with cognitive deterioration due to aging, are therefore not appropriate for use at all levels of ID. These include the following:

(1) The Vineland Adaptive Behavior Scale (VABS) [17];
(2) The Rapid Assessment for Developmental Disabilities, Second Edition (RADD-2);
(3) The Health-Related Quality of Life Short Form Survey (SF-36) [18,19];
(4) The WHO Disability Assessment Schedule (WHODAS 2.0) [20,21,22,23,24].

Because individuals with ID may be at an increased risk of developing dementia more than the general population, especially those diagnosed with Down syndrome, special effort has been made to develop tools for identifying early signs of dementia, such as the Dementia Questionnaire for Learning Difficulties (DLD) [25].

The Adaptive Ability Performance Test (ADAPT) is an instrument that measures adaptive functioning along three domains (conceptual, social, and practical). The ADAPT does not measure language receptivity, the ability to speak, or motor skills. Compared to the Vineland II and 3, the ADAPT measures skills that are relevant in clients with higher functioning levels (i.e., clients with mild IDs or borderline intellectual functioning) [26].

The Adaptive Behavior Assessment System—Second Edition (ABAS-II) [27] was translated into Hebrew and found to be valid and reliable to use with individuals with IDs [28]. However, the shortcoming of this instrument lies in its large number of items [2,10]. The ABAS-II is also the official diagnostic tool of the Disabilities Administration at the Ministry of Welfare and Social Services [29]. Therefore, all the service recipients enrolled within the facility have an official ABAS-II adult version evaluation in their medical files ID (for more details, see [10]).

To summarize during the last decades, various attempts have been made to develop different assessment tools for this population; however, the currently available instruments are not suitable for a quick, practical, online diagnostic screening for continuous use by DSP [2,4,10,11,16]. For the reasons mentioned above, in the first phase of this research, a new Functional Screening Tool for Adults with Intellectual Disabilities (FST-ID) was developed at various adult daycare centers in Israel, carefully maintaining the structure of the existing instruments such as the ABAS-II and in accordance with the ICF model [10].

The new instrument comprises three clusters of functional behaviors: conceptual (CON—5 items), practical (ADL—10 items), and social (SOC—2 items), with the addition of two new items (environmental change and use of assistive devices), and an overall score (GAC) (See Appendix A).

It was finalized after a lengthy, orderly development process involving the DSP and a health-related, multidisciplinary team, with extended experience in working daily with individuals with ID and thus possessing in-depth knowledge of their needs [10].

By the end of phase one (September 2020), the new screening tool was ready to be used in phase two of the research through the mobile phones of the DSPs. The second phase comprised collecting data during the pandemic because a relevant screening tool may assist healthcare providers without increasing their workload.

As a public health crisis, the COVID-19 pandemic caused long-term disruption in the support systems of people with ID across the globe [15,30,31]. Government mandates, often changing overnight, drastically reduced all social contacts, thereby affecting the health and well-being of neurotypical individuals and those with ID. As a result, a reduction in daily support and health services for this group of clients was observed [32]. Research findings suggest a decrease in competencies and social integration and an increase in challenging behaviors, mental health problems, and other comorbid health conditions, e.g., [33,34,35].

In Israel, as in other countries, adults with ID and their DSPs experienced disruptions in their daily routines due to COVID-19. During 2021, only 6.6% of the total number of adults with ID known to the Ministry of Welfare and Social Services in Israel went through a full reassessment, which is mandated by law once every three years [3]. This represents a 40% drop compared to that in the previous years and is attributed to accessibility issues related to the pandemic [36]. The need for such diagnostical reassessments is due to the dynamic nature of ID and is a familiar process routinely performed in many countries, e.g., [11,37].

Furthermore, the DSP, usually with little professional experience and often isolated with their service recipients, became almost the only channel of information regarding the functional and environmental changes experienced by their service recipients [2,15]. For example, at Amichai, (a nonprofit organization that operates daycare centers for adults with ID), only those who lived with their families (mostly those with S/P diagnosis) could regularly attend the centers throughout this period, while those who lived at group homes (mostly those diagnosed at the mild/moderate level) could attend only minimally and partially and were exposed to significant changes regarding previous routines (not attending the day care center) and personnel (due to COVID-19 isolation of both the persons with ID and their service providers).

To diagnose these changes, the Functional Assessment Screening Tool (FST-ID), a new tool that was developed by the authors and found to have high validity and reliability measures [10], was used first as a clinical tool at the start of the COVID-19 pandemic (September 2020–February 2022) [38] and during its last leg (February 2022–June 2022), totaling 22 months of the COVID-19 pandemic by the DSPs.

### Aim of the Study

To summarize the process of constructing and using a new screening tool for mapping the functional and environmental changes experienced by adults with different levels of ID at the start of the COVID-19 pandemic and shortly after it.

## 2. Materials and Methods

### 2.1. Ethics

The study was conducted after obtaining approval from the Ethics Committee of Ariel University (AU-HEA-ML-20200610), the Chief Scientist at the Ministry of Welfare and Social Services, and the managers of the daycare centers at the Amichai nonprofit organization.

The study involved the voluntary participation of direct service providers (DSP) and multidisciplinary staff. Although individuals with IDs did not directly participate, their formal legal guardians within the involved facility signed informed consent forms in accordance with Ariel University’s IRB and Ministry of Welfare and Social Services requirements.

### 2.2. Participants

A total of 37 DSPs participated, the majority of whom were women (78.4%). Their ages ranged from 19 to 55 years (mean = 26.73, SD ± 12.13). All had a daily experience of eight hours with the service recipients for at least three months and members of the direct multidisciplinary team [10,38], who treated 76 adults aged 21 and over, all diagnosed with mild to severe/profound IDs and enrolled within three daycare centers.

The 76 adults, 29 (38.2%) of whom were women, were diagnosed at all levels of ID: mild (N = 31; 41%), moderate (N = 29; 38%), and severe/profound (N = 16; 21%). Their ages ranged from 21 to 71 years (mean = 37.63, SD ± 14.61). According to the official diagnosis (Diagnostic Committees of the Ministry of Welfare and Social Services)*,* as extracted from their personal files, all adults with severe/profound ID (16; 100%) lived with their families, while most adults with moderate ID (22 out of 29, 76%) and mild ID (24 out of 31, 77%) lived in various community residential programs.

All of them attended three daycare centers: 19 (age range: 46–71 years, mean = 60.32, SD ± 7.66) at the senior citizens’ employment day center; 42 (age range: 21–51 years, mean = 30.29, SD ± 6.26) at the therapeutic day center; and 15 (age range: 23–38 years, mean = 29.47, SD ± 4.59) at the supported employment day center.

Most of them presented other comorbidities apart from ID, usually with more than one diagnosis, such as gastrointestinal problems—34 (45%), cerebral palsy—20 (26%), epilepsy—19 (25%), challenging behaviors—16 (21%), vision impairments—19 (25%), autism—9 (12%), and more.

### 2.3. Setting

The study setting is the Amichai nonprofit organization (https://o59769.wixsite.com/amichai-english-site, accessed on 1 September 2022), which operates in Israel and supports service recipients possessing varying levels of ID. Those (aged 21 years or above) who graduate from special education schools at the age of 21 are entitled to visit these settings according to the law in the State of Israel. The organization oversees the integration of these recipients in an array of daycare centers under its management.

#### Changes during COVID-19 and after (September 2020–June 2022)

During the first few months of the pandemic, only the therapeutic center was left open, and only for those who lived with their families. All the other service recipients stayed isolated at their group homes. Gradually, all centers were reopened. However, once a service provider or a service recipient was found to be positive for COVID-19, care providers and recipients were immediately isolated; recipients were accompanied by a care provider. This resulted in endless routine changes, especially for adults with ID who lived in group homes and much less to those who lived with their families and continued to come to the therapeutic day center as they had before the outbreak of the pandemic. The professional team was allowed to work only at the therapeutic center. Therefore, adults with ID who lived with their families were dedicated all the therapeutic hours that were usually spent on both groups (approximately twice, compared to pre-COVID times) [38].

### 2.4. Materials

#### Functional Screening Tool for Adults with Intellectual Disabilities (FST-ID)

The construction and validation of the FST-ID during phase one is described in detail in [10]. By the end of that phase, this new online tool was found to have high psychometric values. The new instrument comprises three clusters of functional behaviors: conceptual (CON—5 items), practical (ADL—10 items), and social (SOC—2 items), with the addition of two new items (environmental change and use of assistive devices), and an overall score (GAC). The answers for each item in the three functional clusters range from 0 to 4, and the total score of the scale ranges from 0 = profound disability to 68 = no disability. Additionally, this scale contains two items: “Environmental changes” and “Using assistive devices,” and an option to add the rater’s subjective comments, as suggested by the WHO [10].

### 2.5. Procedure

In the first phase (September 2019–September 2020), which is detailed in [10], the development process carefully maintained the structure of existing instruments, such as the ABAS-II, in accordance with the ICF model and the AAIDD. The new online tool, the Functional Screening Tool for Adults with IDs (FST-ID), was finalized after a lengthy, orderly development process involving DSPs and a multidisciplinary team possessing experience in working daily with individuals with IDs at Amichai nonprofit organization, and thus having in-depth knowledge of their needs. All tests presented a high degree of reliability of the new instrument’s items and the instrument as a whole [10]. 

In view of the new instrument’s strong psychometric properties, online accessibility, and short completion time (approximately 5 min per service recipient), it could have been considered a useful diagnostic, online tool during phase two of the current research.

In the second phase, during a period of 22 months of the COVID-19 outbreak and shortly after (September 2020–June 2022), the FST-ID was completed by DSPs via their phones for 76 adults with ID who physically attended three daycare centers at four points of time: beginning (September 2020), middle (August 2021), end (February 2022), and post (June 2022) of that period.

The functional score was defined by calculating the mean FST-ID GAC score received by all the service providers who answered for each of the adults with ID at each sampling time point.

## 3. Results

For statistical analyses, SPSS version 28 was used. Descriptive statistical analyses were performed on the demographic data of the service recipients and providers who participated in the study [10,38]. 

### 3.1. Use of Assistive Devices 

**Figure 1:**
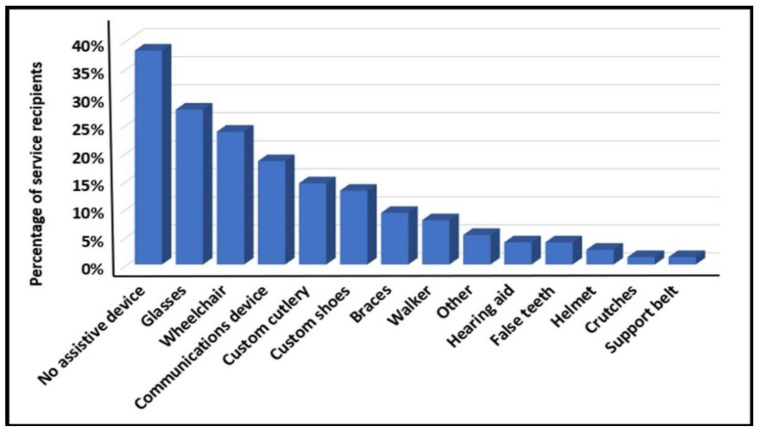
Use of assistive devices (September 2020–end of June 2022 by 76 ID).

Many of them were using assistive devices during the current research (see Figure 1). Fifty-one out of seventy-six (67%) were diagnosed with COVID-19 during the 22 months of the current research (from September 2020–June 2022). Throughout the sampling period, no new accessories were added and the existing ones were partly unused due to a large turnover in the staff and the location of the service recipients. Equipment was forgotten or had broken down, and it was difficult during the epidemic to replace them and to instruct the staff on their correct use.

### 3.2. Phase One: Psychometric Values of the New Tool (FST-ID) (September 2019–September 2020)

The FST-ID presents high psychometric properties and requires less than five min to complete [2]. Criterion validity—high-positive correlations were found between FST-ID and ABAS-II (Gold standard) (*r* = 0.91 *p* < 0.001) scores and between the severity of the ID (according to the official diagnosis per care recipients’ private records) and the FST-ID (*r* = 0.78, *p* < 0.001), implying that the GAC scores were higher for individuals with less severe IDs. High internal consistency was found in the three clusters (CON: α = 0.89; ADL: α = 0.96; SOC: α = 0.62). The scale also holds high intra-rater reliability (Cronbach α = 0.95) and inter-rater reliability values (Cronbach α = 0.97).

The GAC scores are between 0 = total disability to 68 = no disability. A statistical analysis was performed using the ROC (receiver operating characteristics) test. It revealed that the GAC score cutoff point between mild and moderate disability was 50.4 (sensitivity: 87%, specificity 86%, *p* < 0.001), and it was 29.4 between moderate and severe-profound disability (sensitivity: 96%, specificity 94%, *p* < 0.001) (For additional details, see [10]).

### 3.3. Phase Two: Using the FST ID during 22 Months of the Pandemic (September 2020–June 2022)

#### 3.3.1. Functional Changes for 76 Adults with ID

For a better understanding of the interactions between time and various factors (disability level, age, gender, COVID-19, day care, and type of residence) with regard to the functional score, we used a generalized estimating equations test. Time had no significant interaction with age, gender, or COVID-19. 

#### 3.3.2. Functional Changes According to Disability Level

**Figure 2:**
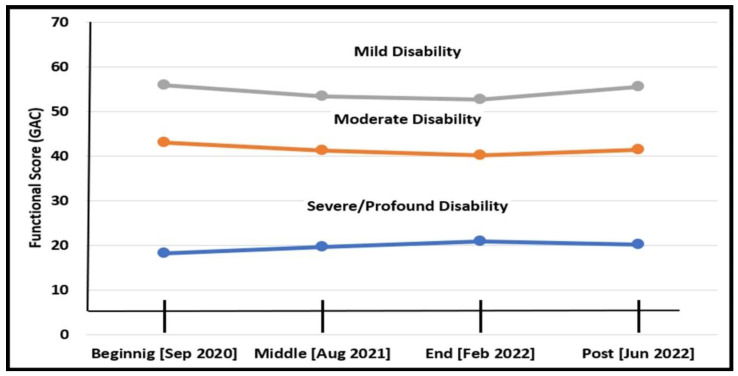
Functional changes for three groups of ID (September 2020–end of June 2022).

As seen in Figure 2, there is a cut-off point between two periods of time: during the COVID-19 period (September 2020–February 2022) and after it (February 2022-June 2022). During the COVID-19 period, the functional score of the severe/profound group improved significantly (Wald Chi-Square = 6.04, df = 2, *p* < 0.05); slightly deteriorated for the moderate group (Wald Chi-Square = 3.37, df = 2, *p* = N.S.); and significantly deteriorated for the mild group (Wald Chi-Square = 10.55, df = 2, *p* < 0.01). After the COVID-19 period, the functional scores for the severe/profound group slightly deteriorated (Wald Chi-Square = 1.46, df = 2, *p* = N.S.); slightly improved for the moderate group (Wald Chi-Square = 0.76, df = 2, *p* = N.S.; and significantly improved for the mild group (Wald Chi-Square = 15.46, df = 2, *p* < 0.001).

#### 3.3.3. Functional Changes According to Type of Day-Care

**Figure 3:**
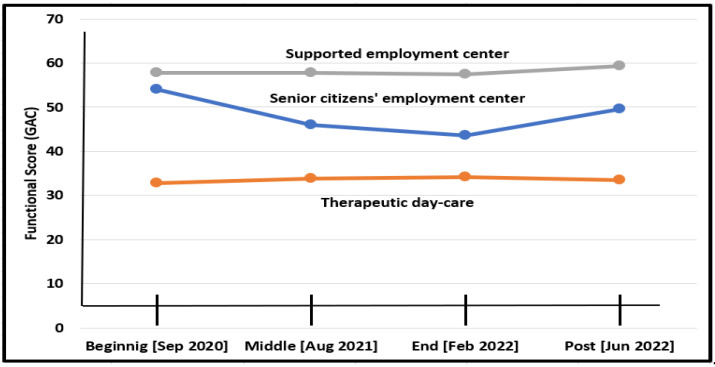
Functional changes for three groups of daycare (September 2020–end of June 2022).

As seen in Figure 3, there was a significant interaction between time and type of day care regarding their functional score. Adults who visited the senior’s daycare center showed a significant functional decrease (Wald Chi-Square = 110.84, df = 2, *p* < 0.001) during COVID-19 and a significant functional improvement after (Wald Chi-Square = 13.72, df = 1, *p* < 0.001). 

The supported employment day center showed no effect during COVID-19 and slightly improved after (Wald Chi-Square = 4.46, df = 1, *p* < 0.05). The therapeutic daycare showed no significant effect during both periods.

#### 3.3.4. Functional Changes According to Type of Residence

There was also a significant interaction between time and type of residence regarding their functional score. Adults who lived with their families in their homes did not present any significant functional change, whereas those who lived in group homes showed a significant decrease in functioning during (Wald Chi-Square = 22.18, df = 2, *p* < 0.001) and a significant increase after (Wald Chi-Square = 5.27, df = 1, *p* < 0.05)

#### 3.3.5. Environmental Changes for 76 Adults with ID

**Figure 4:**
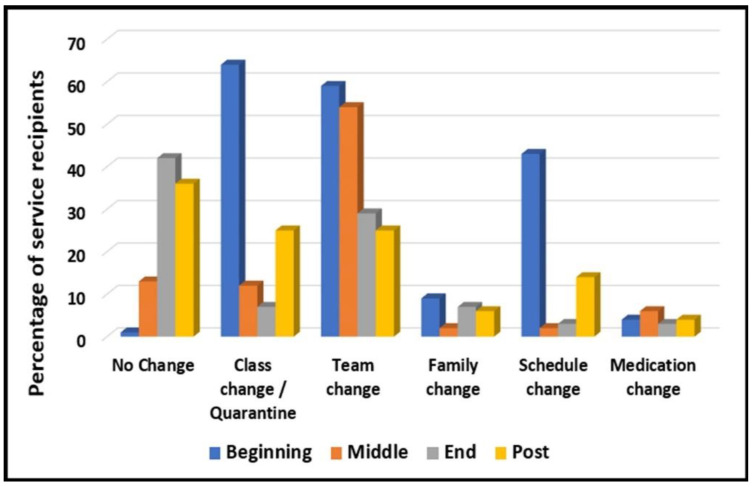
Environmental changes for 76 Adults with ID (September 2020–June 2022).

All 76 adults with ID experienced environmental changes during COVID-19. The highest level of change occurred at the beginning of that time, slightly less in the middle, and minimal change by the end of that period and after its termination (see Figure 4, and for more details, see [38]).

## 4. Discussion

ID is currently viewed by the WHO, AAIDD, and others as a dynamic state that may vary over time [29]. This change stems from the understanding that individuals with ID can improve or deteriorate their functioning in most areas of life through individualized programs and appropriate environmental support when appropriate programs and healthcare services are implemented. However, a reliable diagnostic tool enabling a better differential diagnosis between chronic and new symptoms expressed in changes of the functional state of adults with ID is still lacking. The current article summarizes the process of constructing, validating, and using a new screening tool during the pandemic and shortly after it to map the functional and environmental changes experienced by adults with ID. These data are tremendously important for future planning of health and social services with limited budgets for this population [2,10,39,40,41].

The current research focused on the first stage on the development of a new (online) screening tool comprising quantifiable items. Our findings suggest that the FST-ID can be easily used by DSPs and alert professional therapists to measure the functional and environmental changes in their care recipients, thereby enabling rapid allocation of therapeutic services, with full adherence to the immediate needs of their clients. This is an important goal in a system with limited budgetary resources [4,42].

This is a significant improvement from using lengthy and expensive diagnostic scales mostly used only by healthcare professionals. The development process carefully maintained the structure of existing instruments such as the ABAS-II and was in accordance with the ICF model. It was finalized after a lengthy and orderly development process involving the DSPs, a multidisciplinary team, and content experts. All tests pointed to the high degree of reliability of the new instrument and its items [10].

The FST-ID allows DSPs to actively participate in the decision-making process of care for their service recipients, as suggested by others [16,43], by identifying their functional status with more than 90% accuracy.

The advantages of the new scale include the ease and speed with which it can be completed and the fact that its domains were adapted specifically to adults at all levels of ID, regardless of the degree of disability, often with multiple comorbidities, who attend daycare centers. Moreover, the instrument’s accessibility allows for the collection of information from many DSPs concurrently, and, consequently, offers a comprehensive, real-time description of each service recipient’s condition, thereby assisting the differential professional diagnosis between the chronic comorbidities to new symptoms such as post COVID 19 symptoms, by healthcare professional as was suggested by others [44].

Another advantage is the fact that the new instrument has an online version for mobile phone use, preparing healthcare of this population for a digital world, in line with the increasingly popular approach of telemedicine and e-health, as observed by others [10,15,45,46].

Furthermore, with the decline in the current wave of the corona virus, it is necessary to continue follow-up with various screening tools. People with an ID following acute COVID-19 may benefit from a follow-up to help identify symptoms of long-COVID and any additional care needs. In addition, it will make it easier for the medical teams to diagnose whether there are late symptoms of the COVID-19 virus or other medical symptoms due to the comorbidity that characterizes this population [44], such as long-term and multiple time over dose of psychiatric medications [39,47].

According to the ICF model [40], the environment has a tremendous effect on individual function and participation. This pandemic has added additional environmental limitations to an already vulnerable and limited population of individuals with ID due to their disability [48]. We assumed that to prepare for emergencies such as pandemics and other crises, we must follow-up with appropriate diagnostic tools and reach rapid conclusions that will enable the construction of appropriate treatment plans for individuals with ID despite their communicational, physical, and behavioral limitations. In the first phase of the current research, we found that the new tool, the FST-ID, enables quick and knowledgeable informed healthcare diagnostic and functional evaluation decision-making.

In the second phase of this research, the FST-ID was explored as an ongoing clinical tool, enabling the determination of the impact of environmental changes on individual functional abilities during the 22 months of the outbreak and fading of the COVID-19 pandemic for 76 adults at all levels of ID at four time points: beginning, middle, end, and post-COVID-19.

As expected, the entire group of 76 adults with ID experienced environmental changes. These changes occurred mostly at the beginning of the year (September 2020), when routines were severely damaged. Gradually, the environmental changes became milder. Within the duration of the research, two main periods (during COVID-19 and after the pandemic) occurred with a turnover of the functional trends for each of the three disability groups at the cutting point (February 2022).

While there was a significant reduction in functional abilities of the M/M groups suffering most changes due to the new regulations as reported by others as well [15,33,49], during COVID-19, there were significant improvements by the S/P group, mainly due to intensified health-related intervention [10]. After the pandemic, when the regular schedule and therapeutic hours gradually returned to pre-COVID times, the S/P group deteriorated, while the M/M improved. The senior’s daycare group was most affected by these changes, probably due to the combined effect of environmental changes: all of them lived in group homes (where there was a functional deterioration during the pandemic and improvement shortly after it) with personal factors—their other comorbidities due to their quick aging with the evidences already well-documented of the effect of the pandemic on the aging population in general [50]. Before the pandemic, the adults in this study, like adults with IDs in other countries, visited daycare centers and lived either at their family’s home or at various community group homes, with support from family members or paid service providers [3,34,51]. The daily program of constructed routines and activities established a stable framework consistently supporting their well-being and functional abilities [34,52].

During the first months of the pandemic, due to repeated lockdowns imposed by governmental regulations, those routines and habits dramatically changed: only one daycare center—the therapeutic center—was left open and continued to provide services to adults with ID who lived with their families, all of whom were individuals diagnosed with S/P ID. Most individuals diagnosed with M/M ID remained at their group homes and experienced a significant reduction in therapeutic services and complete change in daily routines because they were not allowed to meet with their families and other group home members, according to governmental regulations. Moreover, this period was also characterized by several personnel changes, including daily unexpected changes and a reduction in the number of care providers due to exposure to COVID-19 (by the care providers themselves and between the group home members). This situation caused a further effect: unexperienced DSPs were left alone with no professional supervision at the group homes where the M/M ID lived. This phenomenon was also reported by authors from different countries [15,31,49,53].

During this period, with the intention of reducing infections by COVID-19, the professional staff worked only in the therapeutic center. As a result, the S/P group gained approximately twice as many health-related professional therapeutic hours (intensified physiotherapy, smaller communication, and art therapy groups) compared to pre-COVID-19 days and presented statistically significant functional improvement [38].

At the end of the COVID-19 period, when going back to previous routines and therapeutic services, the group diagnosed at M/M returned to almost the same functional state they presented at the beginning of the pandemic. At the same time, the S/P group showed the opposite trend due to a reduction in professional treatment hours, which were now given to all groups of disability before the outbreak of the pandemic.

The main finding of this work—the dynamic nature of adult ID (expressed by functional changes due to environmental COVID-related changes)—adds evidence-based support to the ICF biopsychosocial model. As we have witnessed, the environment can influence the functional ability of adults with ID at all levels. It can be improved or deteriorated by routine and social barriers, as well as by reducing or intensifying therapy [2,38,54].

The new screening tool FST-ID used in this research offers a quick way to map functional and environmental changes at all levels of ID and provides a reliable diagnosis of the current level of ID at approximately 90% accuracy and without any costs by their DSPs. The routine use of such a tool may reduce existing costs spent on expensive diagnostic procedures and allows for better distribution of healthcare services for this population.

For example, the Israeli Ministry of Welfare spends 12.5 M NIS (3.9 M USD) annually on re-assessments for a relatively small portion of adults with ID. In comparison, only 3.5 M NIS (1.1 M USD) from the welfare budget is spent on assistive devices for the entire ID population in the country [36]. An official response to a request regarding budgetary expenses by the Israeli Ministry of Welfare and Social Services (IMWSS) was sent by the authors (August 2021), with a reply sent by E-mail, September 2021. This small sum is due to low budgetary availability by the government and due to a shortage of appropriate assessment tools and accessibility difficulties. Another issue which might be raised regarding the use of the FST-ID is the socioeconomic level of families caring for a person with ID. Payments are only partially funded by the national Israeli insurance. Caring for the person with ID becomes more challenging, when the child becomes an adult with their added co-morbidities [55].

In the current research, all families were classified as being at a medium–high socioeconomic level and, therefore, were able to cope with their child’s growing needs. However, the authors believe that when it comes to families ranked at low socioeconomic situation, this can become a hindrance for proper care. In such situations, as well as in peripheral areas where medical support is less than optimal. The regular use of the FST-ID as a rapid alert tool is even more essential.

During this research, by using the FST-ID, it was found out that service receivers relatively utilize several assistive devices. Most of them were not changed or not regularly used partially due to less accessibility during the pandemic, repairs incurred, changes in the regular surrounding where they were used, etc.

As was advised by others, e.g., [13], policy-makers should ensure that money devoted to individuals with ID is allocated in a rational, equitable, and cost-effective manner, i.e., for renewing and adding new assistive devices according to the dynamic needs of these service receivers.

Furthermore, we assume that to prepare for emergencies such as pandemics and other future crises, where changes might be swift, we must follow-up with appropriate screening tools and reach rapid and consensus-based conclusions that will enable the construction of appropriate treatment plans for individuals with ID despite their communicational, physical, and behavioral limitations. Given the likelihood of further restrictions that might be imposed, clear policies and advice for older people in general, especially those with ID, should be prioritized to ensure better access to health services. 

## 5. Conclusions

This research summarizes the complete process of constructing, validating, and clinically using a new tool, the FST-ID. This screening tool enables a low-cost, quick, and reliable mapping of the functional and environmental changes experienced by adults at all levels of ID.

Our two main findings suggest that during the outbreak of COVID-19, the function of adults diagnosed with M/M levels of ID deteriorated due to changes in their daily routines, reduction in therapeutic interventions, and social isolation, and those who used to visit the senior’s daycare experienced the largest functional changes compared to the other daycare centers. Subsequently, the S/P group maintained their routines and gained increased health-related therapeutic interventions. During the fading of COVID-19 and the return to regular routines, those trends reversed. The current findings support the perception represented by the WHO biopsychosocial model, suggesting that individuals with ID at all functional levels may improve or deteriorate in their functional abilities according to environmental changes.

## 6. Limitations of the Study

The current study was carried out on a relatively small number of adults with ID: 76 individuals across three groups of ID. We emphasize that all our results should be considered with additional caution owing to the small sample size and relatively short period of sampling. Larger and more carefully controlled samples might facilitate a better understanding of related changes in functional abilities. The study was conducted during the pandemic and only for a short period after it. We, therefore, recommend using the FST-ID for adults with ID for long and continuous periods of time, thereby enabling a better differential diagnosis between chronic and new symptoms in this population. This might also help in more effective future planning by stakeholders on how to reallocate budgetary expenses for this population. 

## Data Availability

The data that support the findings of this study are available on request from the corresponding author. The data are not publicly available due to privacy and ethical restrictions.

## Appendix A

Name of service recipient __________________________

Date: ___ Completed by: _____ How long have you been working with the service recipient? ___

Each rectangle contains items that describe functional behavior. Circle the item that is most suitable for the service recipient in your opinion.

**Table:**

Communications and Academics (CON)	Score
Communications: communicates well with everyone (4), communicates reasonably well with some people (3), maintains limited communication with few people (2), rarely communicates with the human environment (1), does not seem to communicate (0).	
Language and speech: fluent and coherent (4), short but clearly understandable sentences (3), most speech is not clear (2), single words, many unclear (1), does not seem to speak at all (0).	
Writing (by hand or with the assistance of a switch, a spelling board, etc.): complete, entire stories (4), writes sentences (3), writes words (2), copies few words or letters (1), does not write (0).	
Comprehension: understands everything (4), understands daily/related issues (3), understands most instructions (2), understands few/basic instructions (1), does not understand instructions (0).	
Reading: reads short stories (4), reads short sentences (3), reads at least 5 words (2), identifies letters (1), not able to read (0).	
**Practical activities of daily living (ADL) and self-care**	
Mobility: walks, sits, and lies down independently (4), is mostly independent but uses walker or wheelchair (3), needs mild supervision in transitions (2), needs extensive support in transitions (1), needs complete support in all ADL (0).	
Falls: Walks independently without any falls in the past year (4), lost their balance last year but did not fall (3), fell several times this year (2), fell several times this week (1), needs constant support/supervision to prevent falls (0).	
Daily activities (preparing a beverage, organizing a desk or classroom, throwing out the garbage, putting things back in their place): completely independent (4), needs mild supervision and verbal direction during ADL (3), need physical assistance in a small number of activities (2), needs assistance in most activities (1), needs full support to perform these activities at all (0).	
Leisure activities (draws, flips pages through a newspaper, strings beads, plants a plant): completely independent (4), needs only supervision and light verbal direction (3), needs a little physical and mostly verbal support (2), needs extensive physical & verbal support to participate (1), is unable to perform these activities at all (0).	
Eating and drinking: independent (4), needs food to be cut (3), needs mild assistance and/or mostly independent with special cutlery (2), needs to be fed completely (1), enteral feeding only (0).	
Food consistency: ordinary (4), cut into small pieces (3), chopped (2), blended (1), food blended and beverage with thickening agent (0).	
Mealtime behavior: ordinary (4), mild supervision only (3), custom eating environment and group supervision (2), staff member provides personal supervision due to challenges (1), does not eat in the setting (0).	
Toileting: completely independent (4), independent but needs verbal reminders (3), needs assistance only in wiping or during menstrual period (2); partial control of urination or soiling (may alert caregiver) (1), no control at all/wears diapers continuously (0).	
Dressing: completely independent (4), needs mild assistance (mostly verbal) (3), needs moderate assistance (with shoelaces, buttons, and zippers) (2), mostly not independent (1), does not dress independently (0).	
Self-care and hygiene (washes hands, awareness of deodorant, menstrual products, bad breath, etc.): independent (4), needs mild assistance/reminders (3), needs moderate assistance (2), does almost everything with assistance (1), full support (0).	
**Social and emotional (SOC)**	
Participation in activities: full (4), generally participates (3), sometimes participates (2), rarely participates (1), does not participate in any activity (0).	
Challenging behavior (shouts, spits, pinches, pushes, hits, undresses, insults): regular behavior (4) rarely (3), sometimes (2), most of the time (1), all the time (0).	
**Total score for all clusters (GAC)**	
**Use of assistive devices**	
Use of assistive device: wheelchair, walker, crutches, helmet, custom/medical shoes, support belt, braces, corset, communications device, glasses, hearing aid, false teeth, custom cutlery, other.	
**Environmental changes**	
Did any of the following occur recently: Change in staff, change in family, change in class/place of residence/isolation, change in schedule, change in medication. Other (specify): _____________.	
